# Emergence and evolution of epizootic hemorrhagic disease virus in the Mediterranean region: spatio-temporal dynamics and epidemiological insights

**DOI:** 10.3389/fvets.2025.1569244

**Published:** 2025-07-29

**Authors:** Marwa Arbi, Emna Harigua-Souiai, Mariem Hanachi, Imen Larbi, Melek Chaouch, Dorra Rjaibi, Mohamed Fethi Diouani, Alia Benkahla, Oussema Souiai

**Affiliations:** ^1^Laboratory of Bioinformatics, Biomathematics and Biostatistics, LR16IPT09, Institut Pasteur de Tunis, University of Tunis El Manar, Tunis, Tunisia; ^2^Laboratory of Molecular Epidemiology and Experimental Pathology-LR16IPT04, Institut Pasteur de Tunis, Université de Tunis El Manar, Tunis, Tunisia; ^3^Laboratory of Epidemiology and Veterinary Microbiology, LR16IPT03, Institut Pasteur de Tunis, University of Tunis El Manar, Tunis, Belvédère, Tunisia; ^4^Laboratory of Molecular Microbiology, Vaccinology and Biotechnological Development, University of Tunis El Manar, Tunis, Belvédère, Tunisia; ^5^Higher Institute of Biotechnology of Béja, University of Jendouba, Beja, Tunisia

**Keywords:** phylogeny EHDV, phylogeographic analyses, EHDV, tMRCA, Mediterranean basin EHD

## Abstract

**Background:**

Epizootic Hemorrhagic Disease Virus (EHDV) is an arbovirus, transmitted to wild and domestic ruminants through Culicoides biting midges. Since 2006, high morbidity and mortality cases of EHDV have been reported among cattle and deer populations in several Mediterranean countries. The temporal and geographic origins of these incursions remained unclear. In this study, we aimed to investigate the evolutionary history of EHDV in the Mediterranean region and highlight the epidemiological features of viruses in relationship with genetic diversity and viral ecology.

**Methods:**

We extracted from GenBank the EHDV VP2 and VP5 segments isolated in the mediterranean region during the period 2006 to 2023 and blasted them to obtain a final dataset of 68 and 91 nucleotide sequences. Using these datasets, we conducted a Bayesian phylodynamic analysis, which inferred discrete models of “geographic origin,” “Serotype” and “Host” by employing the BEAST package.

**Results:**

RSPP and TMRCA analyses showed that the Mediterranean EHDV has as ancestral root the North America strains that circulated in the 17^th^ century. Our study suggested that the first EHDV incursions in the Mediterranean region started in France and Tunisia during the 1800s. The latter countries were epicenters of EHDV in the region. Significant transition routes (BF>3) were detected revealing virus transmission between North African and European countries. Serotype model study revealed VP5 multiple inter-serotype events involving serotypes 1, 2, 6, 7, and 8 with high statistical support (BF>100). Significant virus transmission was detected for Cattle-deer and Culicoides-Cattle transition routes.

**Conclusion:**

The virus transmission was intense between North African and European countries of the Mediterranean region. EHDV spread in this region seems to be influenced mainly by vector/host distribution and abundance, ruminants' trade and prevailing winds.

## Introduction

Epizootic Hemorrhagic disease (EHD) is an arthropod-borne transmitted viral disease affecting wild and domestic ruminants, transmitted by Culicoides biting midges (1). Deer and cattle populations are the most affected hosts, developing mild to severe clinical signs of EHD (1). In cattle, EHD leads to symptoms such as fever, loss of appetite, lameness, and respiratory distress, with a fatality rate of < 1%. Most affected animals recover fully after a few days or weeks with rest and appropriate treatment. In contrast, the disease is more severe in deer, where the mortality rate can exceed 90%.

This disease is caused by the double-stranded RNA Epizootic Hemorrhagic disease virus (EHDV) that belongs to the *Sedoreoviridae* family. The EHDV genome is constituted of 10 segments (2, 3), encoding for seven structural proteins (VP1-VP7) and four non-structural proteins (NS1, NS2, NS3/NS3A and NS4) (4, 5). The most variable proteins of the virus are the outer capsid proteins VP2 and VP5 which are encoded by segments 2 and 6, respectively (1, 4). These proteins are important for viral infectivity by being implicated in viral attachment and entry of the virus into the host cell (4). Genetic and antigenic evolution of EHDV is mainly provided by point mutation and reassortment mechanisms that promote viral fitness and genetic diversity (6). At present, eight serotypes (EHDV1-2, EHDV4-8 and EHDV-10), genetically distinct, have been identified worldwide and are characterized by variable geographic distribution (7, 8).

EHDV was detected for the first time, in 1955, in the USA where hundred cases of death were reported among the white-tailed deer population (9). Since then, the virus has spread in North America, Asia, Europe, Africa, and Oceania, causing significant morbidity and mortality. In 2008, the World Organization for Animal Health (WOAH) included the EHD in the list of notifiable diseases since the emergence of virus serotypes with increased pathogenicity in the Mediterranean region (1). Indeed, EHD became a notable concern in this region, following the first incursion of EHDV serotype 6 (EHDV-6) in 2006, which caused substantial mortality and economic damage across southern Mediterranean countries, notably Morocco, Algeria, and Tunisia (10–12). Although milder cases of EHDV-6 were reported in Tunisia in 2012–2013, a more severe outbreak occurred between September and November 2021. This outbreak, driven by serotype 8 (EHDV-8), rapidly spread across Tunisia, affecting over 200 cattle and wild deer populations (11). EHDV-8 marked the first major circulation of the virus in North Africa and concurred with similar outbreaks in southern Europe, particularly in the Iberian Peninsula (Spain and Portugal), Italy and France in 2022–2023 (12–14).

In Mediterranean countries, EHDV emergence is mainly reported between August and November, aligning with peak Culicoides midge activity. However, the extension of milder winters and prolonged warm season lead to vector survival, breeding cycles, and viral replication (15, 16). This highlights the need to improve epidemiological surveillance and a better characterization of diffusion patterns favoring the epidemiological status. Present monitoring strategies mainly depend on serological methods, including ELISA and virus neutralization assays, alongside RT-PCR techniques used for virus identification and genotyping (8). However, regional harmonization of surveillance protocols is still limited, and timely molecular characterization is often only initiated during or after clinical outbreaks (3). Controlling vectors, especially Culicoides species, continues to be a crucial strategy, involving measures such as habitat management, application of insecticides, and safeguarding animals during periods of high vector activity (17).

The economic consequences of EHDV outbreaks in the Mediterranean basin were significant. In fact, the virus emergence in the region caused losses due mainly to illness, mortality and trade limitations of domestic livestock (1). Moreover, EHDV poses a threat to wildlife, particularly deer population, which could have lasting effects on biodiversity and ecosystem health (18, 19). No commercial vaccine anti-EHDV is available for use in the Mediterranean region, which complicates the control of EHDV emergence and re-emergence among domestic and wild livestock. In response, Mediterranean countries have strengthened vector surveillance, veterinary monitoring, and research on potential vaccines to curb the virus's spread (20). These efforts have led to the development of new diagnostic tests (21) and a deeper understanding of the virus's genetics.

Understanding how viruses like Epizootic Hemorrhagic Disease Virus (EHDV) spread and evolve is essential for staying ahead of outbreaks. Phylodynamic studies, which combine genetic data with information about time and location, offer a powerful way to uncover these patterns. Surprisingly, despite the growing concern about EHDV in the Mediterranean region, no such study has been done until now. By using this approach, our study focuses on the temporal and spatial dynamics of EHDV spread in the Mediterranean region, examining evolutionary aspects of the VP5 and VP2 segments to shed light on ancestral reassortments and host-virus interactions specific to this region.

## Materials and methods

### Dataset preparation

We queried full length nucleotide sequences of segments 2 and 6, encoding for VP2 and VP5 proteins of Mediterranean EHDV strains from Genbank. We extracted 47 and 26 sequences, corresponding respectively to VP2 and VP5 segments, of EHDV that circulated in the Mediterranean countries from 2006 to 2023. The dataset of VP2 segment is composed of EHDV strains from Tunisia (*n* = 14), Algeria (*n* = 1), Morocco (*n* = 1), France (*n* = 7), Italy (*n* = 2), Spain (*n* = 11) and Israel (*n* = 11). The dataset of the VP5 segment contains strains from Tunisia (*n* = 14), France (*n* = 4), Italy (*n* = 3) and Israel (*n* = 6; Supplementary Table S1).

To enlarge the dataset, we conducted nucleotide BLAST NCBI searches (https://blast.ncbi.nlm.nih.gov) using Tunisian sequences as queries. This approach enabled the identification and inclusion of additional homologous sequences from global EHDV strains available in GenBank. The final datasets are composed of 68 sequences of VP2 segment and 91 sequences of VP5 segment, which belong to serotypes EHDV-1, EHDV-2, EHDV-6, EHDV-7, EHDV-8 and EHDV-10 isolated from cattle, deer and culicoides (Supplementary material 1). The information about virus hosts was not available for some strains isolated during 1955–1983 and was labeled as “Unknown” in the metadata table (Supplementary material 1). They also included EHDV strains from the USA, Canada, Ecuador, Australia, Trinidad and Tobago, South Africa, Nigeria, China and Bahrain.

### Phylogeny and phylogeographic analyses

VP2 and VP5 segments were aligned by the online version of MAFFT (https://mafft.cbrc.jp/alignment/server/index.html). We used the Bioedit program (22) to adjust the lengths of the segments and verify their reading frame. To check for potential recombination among EHDV sequences, VP2 and VP5 nucleotide sequence alignments were analyzed using RDP5 software. A recombination event was considered as significant, if supported by at least 4 detection methods within RDP5 and had a Bonferroni corrected *p*-value < 0.05 (23). No significant recombination events were detected in VP2 and VP5 nucleotide sequence alignments.

A Maximum Likelihood (ML) tree was generated by the PhyML (24) program using the GTR+G model which was selected as the best-fit model using the program Smart Model Selection (SMS) (25). To explore the temporal structure of the prepared dataset, the ML trees were analyzed by using TempEST (26).

To infer a time scaled Bayesian maximum-clade-credibility (MCC) tree for the VP2 and VP5 segments, Markov Chain Monte Carlo (MCMC) analyses were performed by running BEAST v1.10.4 (27) analysis for more than 100 million sampling iterations. These analyses were implemented employing Bayesian skyline model which was chosen for its flexibility in modeling the complex dynamic histories, especially for RNA viruses (28). For the clock model, the Path Sampling (PS) and Stepping Stone (SS) method was performed to select the best-fit model between strict clock model and uncorrelated lognormal relaxed molecular clock. According to Bayes Factor (BF) calculated from the resulting values of log marginal likelihood, the best-fit model was the uncorrelated lognormal relaxed molecular clock for VP2 (BF>127) and VP5 (BF>6) aligned nucleotide sequences. The discrete trait diffusion models analyzed in this study were “Country,” “Host type” and “Virus serotype.” Bayesian Stochastic Search Variable Selection (BSSVS) with a symmetric phylodynamic model were applied for each model to determine the BF. This model was demonstrated by Alkhamis et al. (29) as the best-fit model to study host and geographic models of the evolutionary phylodynamics among another orbivirus which is the Bluetongue virus.

We employed TreeAnotator v1.10.4 (27) to generate the MCC trees by removing the first 10% of samples from each MCMC chain. Figtree v1.4.3 software was used to visualize the MCC trees and the posterior probabilities (pp) of tree nodes (http://tree.bio.ed.ac.uk/software/figtree/). The estimation of Time to Most Recent Common Ancestor (TMRCA) and the Root State Posterior Probabilities (RSPPs) was performed by using Figtree v1.4.3.

To calculate the BF, SpreaD3 was used and a BF > 3 was considered as a threshold for statistical assessment of the transition routes between countries, serotypes and hosts (30). We constructed spatio-temporal dynamics for VP2 (Supplementary material 2) and VP5 by generating KML files using the SpreaD3 program (Supplementary material 3). Then, we visualized them on the 3D maps of Google Earth Pro (https://www.google.com/earth/versions/).

### Sampling bias evaluation

A sensitivity analysis was conducted using the “randomized tip swap” method to evaluate potential sampling biases in each discrete trait diffusion model (geographic origin and serotype). The generated XML files with BEAST 1.10.4 were modified to include the tip swap analysis in the operator block, and each MCMC run was carried out for 100 million iterations, with sampling every 1,000 states. Effective sample size (ESS) values >200 were achieved for the estimated parameters. To ensure an unbiased sample, the transition rates for both principal and tip swap analyses should be similar, with the difference between them not exceeding one transition. Large difference suggests that the results are influenced by the sampling procedure rather than the genetic data itself (31).

## Results

### Temporal and geographic origins of epizootic hemorrhagic disease virus in the Mediterranean region

**Figure 1:**
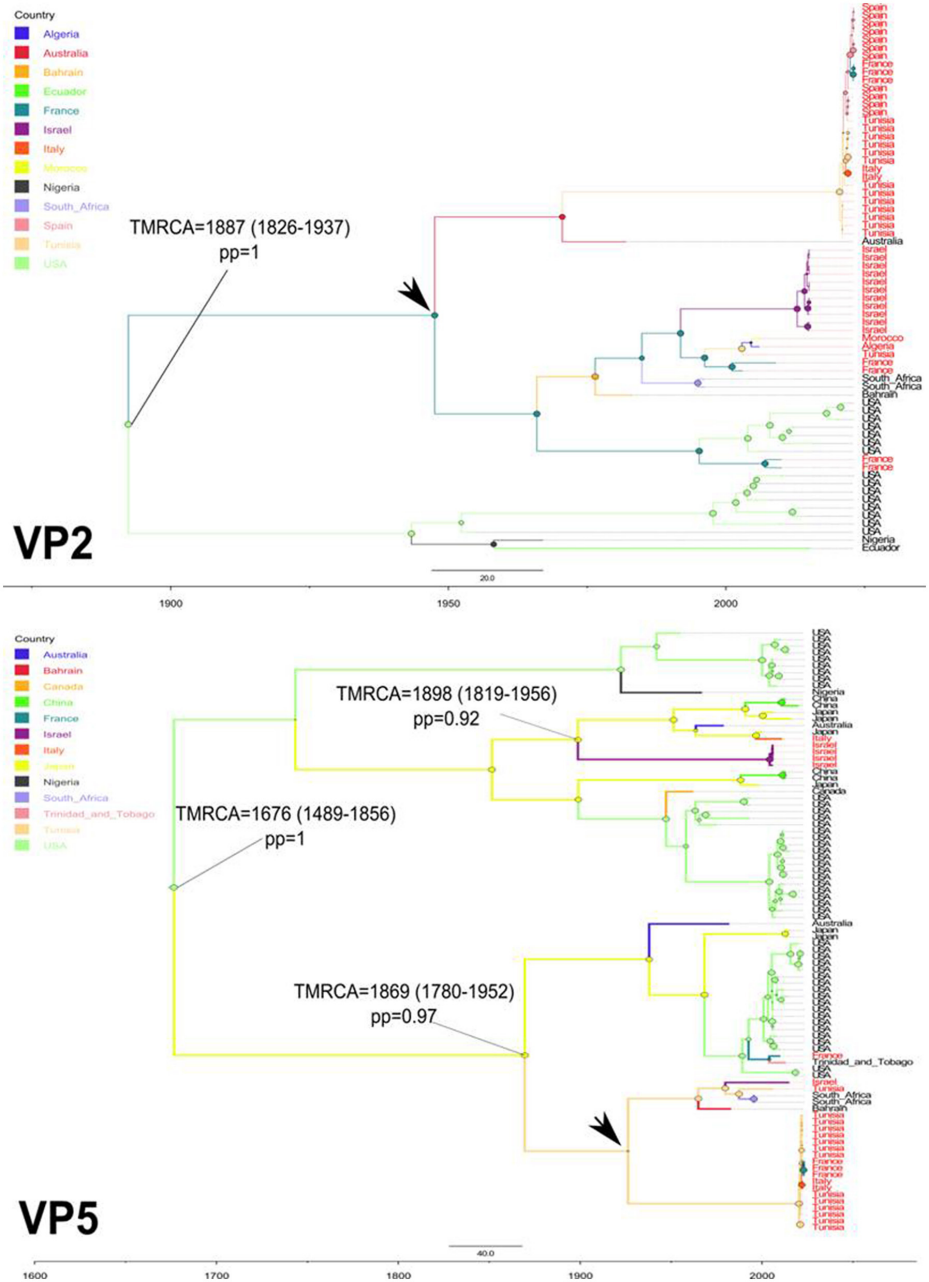
Time-scaled Bayesian MCC trees expressing the geographic origin as discrete trait for VP2 and VP5 segments of EHDV that circulated in the Mediterranean region (Spain, France, Italy, Tunisia, Algeria, Morocco and Israel) during 2006–2023. TMRCA, their 95%HPD and posterior probabilities (pp) are indicated on the nodes of tree root and the ancestral geographic origin of the Mediterranean region EHDV strains. Black arrows indicate the geographic locations marking the first introduction of EHDV in the Mediterranean region.

**Figure 2:**
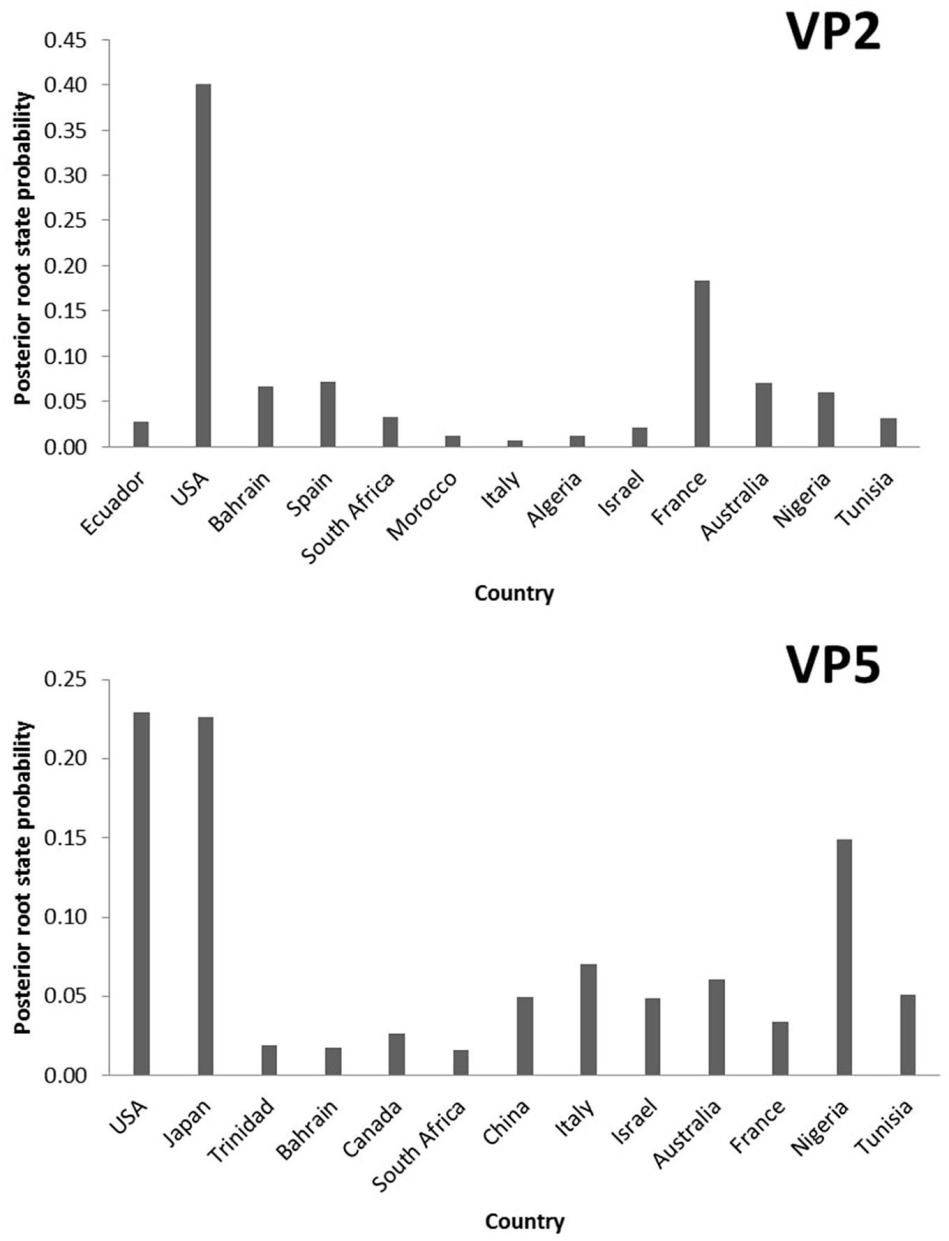
Posterior root state distribution of the “Geographic origin” model for VP2 and VP5 segments of EHDV.

To explore the temporal and geographic origins of Epizootic Hemorrhagic Disease Virus (EHDV) in the Mediterranean region, Bayesian scaled MCC trees inferring ‘virus geographic origin' trait were constructed based on the datasets of VP2 and VP5 segments. The temporal origin analysis showed that The TMRCA of VP2 ancestral root is estimated as 1,887 [95%HPD: 1,826–1,937] (pp = 1). For the VP5 segment, our analysis estimated the TMRCA to 1,676 [95%HPD: 1,489–1,856] (pp=1; Figure 1). The spatial reconstruction indicated that the United States of America (USA) has the highest root state posterior probability (RSPP = 0.40) for the VP2 segment. Concerning the VP5 segment, the highest root state posterior probability is shared equally between the USA and Japan (RSPP = 0.23; Figure 2).

The TMRCA estimation indicated that the first point of EHDV introduction in the Mediterranean Basin was 1,887 [95%HPD: 1,826–1,937] for the VP2 segment (pp = 1). The VP5 segment analysis showed two primary entries of virus in the Mediterranean countries, occuring in 1,869 [95%HPD: 1,780–1,952] (pp=0.97) and 1,898 [95%HPD: 1,819–1,956] (pp = 0.92; Figure 1). The MCC tree and RSPP analyses of the VP2 segment showed that France (RSPC = 0.18) could be a primary source of EHDV in the Mediterranean region (Figures 1, 2). For VP5, Italy (RSPP = 0.07) and Tunisia (RSPP = 0.05) constituted probable introduction points of EHDV in the Mediterranean region (Figures 1, 2). Based on TMRCA and MCC tree analysis, Tunisia could be the oldest point of virus entry in the Mediterranean region.

### Spatio-temporal dynamics of EHDV spread in the Mediterranean region

**Figure 3:**
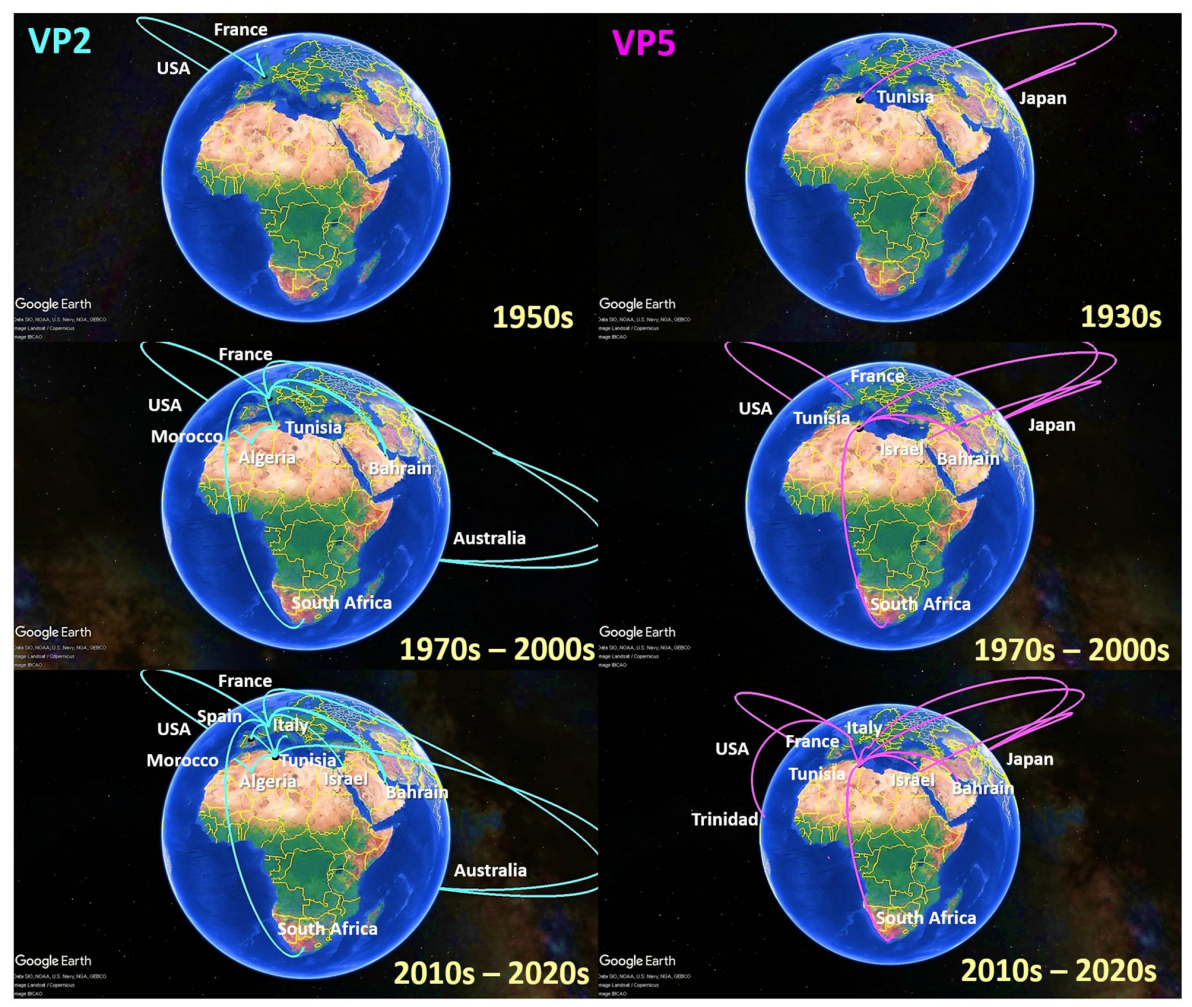
Spatio-temporal dynamics of VP2 and VP5 genes describing the Epizootic Hemorrhagic Disease Virus (EHDV) history spread in the Mediterranean region (Spain, France, Italy, Tunisia, Algeria, Morocco and Israel).

**Figure 4:**
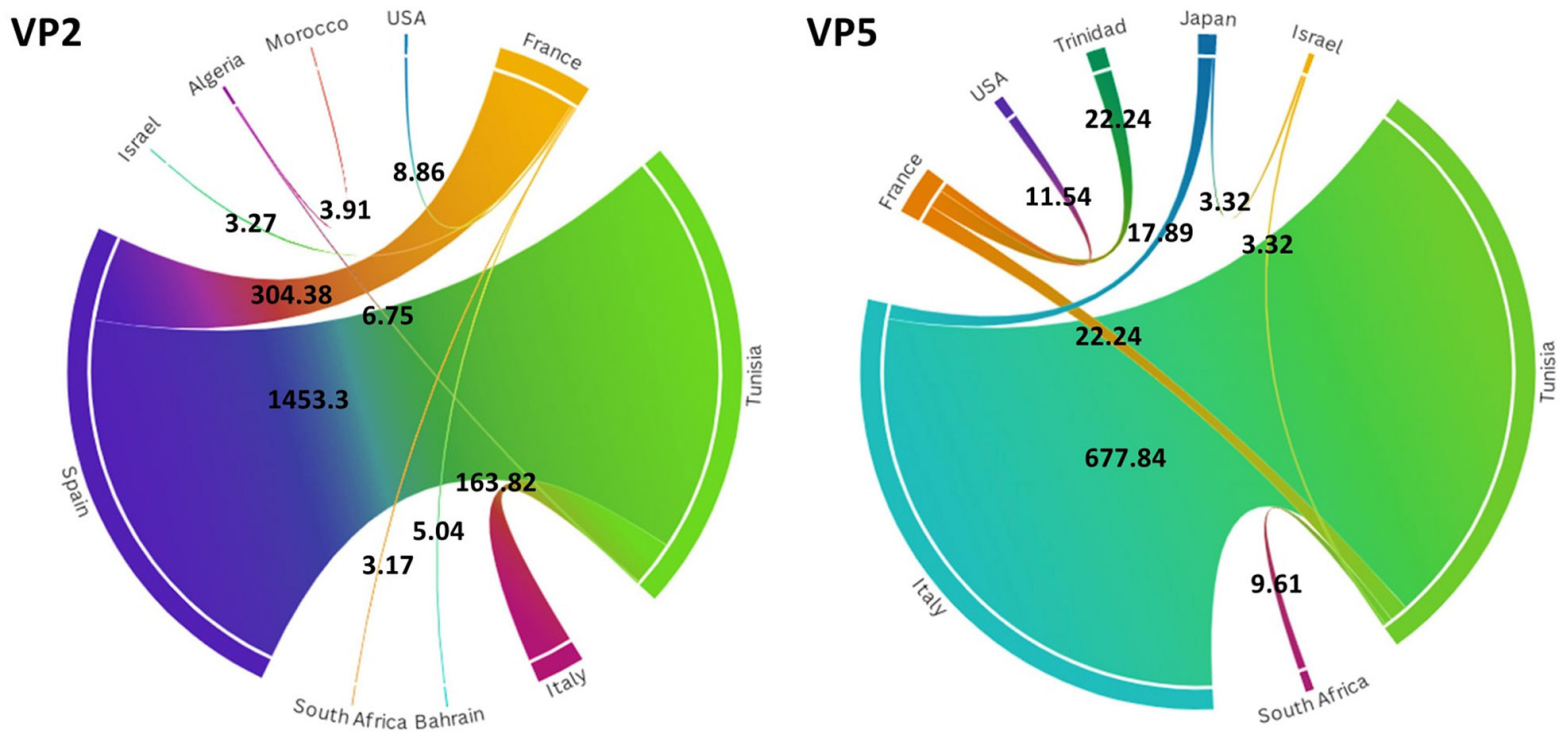
Chord diagrams for VP2 and VP5 segments of EHDV showing the transmission routes of virus between countries with their Bayes factors. BF values are displayed on the chords. Chord width is proportional to BF.

To investigate the temporal and spatial evolutionary dynamics of EHDV in the Mediterranean region, Bayesian networks were generated for VP2 and VP5 segments and mapped on 3D maps. Our latter analysis (Figure 3) suggested that the EHDV started its circulation in the Mediterranean region by affecting France and Tunisia after virus transmission from East Asia (Japan) in the 1930s and North America (the USA) in the 1950s. However, solely, the VP2 transition route linking the USA to France is statistically valid (BF = 8.86), whereas, the VP5 transition route of Japan–Tunisia was insignificant (BF = 0.81; Figure 4).

Since the 1970s, the EHDV infections have extended from France and Tunisia to non-Mediterranean regions notably Australia and Bahrain in West Asia. BF determination for these transition routes provides support only for France–Bahrain in VP2 spatio-temporal dynamics construction (BF = 5.04). The other transition routes linking France to Australia and Tunisia to Bahrain were invalid by BF [VP2: France–Australia (BF = 1.79); VP5: Tunisia–Bahrain (BF = 2.87)] (Figures 3, 4).

The period 1990s−2000s was especially characterized by the virus apparition in other Mediterranean regions such as Algeria, Morocco and Israel with significant BF values (BF > 3). The virus propagated in the neighboring North African countries [VP2: Tunisia–Algeria (BF = 6.75); Algeria–Morocco (BF = 3.91)], Europe [VP5: USA–France (BF = 11.54)] and the Middle East [VP5: Japan–Israel (BF = 3.32)]. In VP2 and VP5 spatio-temporal dynamics constructions, significant Tunisian and French Transition routes with South Africa were also observed during this period (BF = 3.17–9.61; Figures 3, 4).

From the 2010s until the early 2020s, Tunisia and France played the role of epicenters for EHDV spread not only in the Mediterranean region, but also in non-Mediterranean countries. Firstly, EHDV strains spread from France and Tunisia to Israel and Trinidad (BF = 3.32 for Tunisia–Israel; BF = 3.27 for France–Israel; BF = 22.24 for France–Trinidad). Then, the first EHDV incursions in Italy and Spain have occurred. All transitions related to these countries have a high statistical support (BF > 3) where we observed virus transmission from Japan [VP5: Japan–Italy (BF = 17.89)]; France (BF = 304.38 for France–Spain) and Tunisia (VP2: BF = 1,453.30 for Tunisia–Spain; VP2/VP5: BF = 163.82–677.33 for Tunisia–Italy). The virus was also transmitted to France from Tunisia during the 2020s and this transition route was highly significant in the VP5 spatio-temporal dynamics construction (BF = 22.24; Figures 3, 4).

### Host and serotype phylodynamics

**Figure 5:**
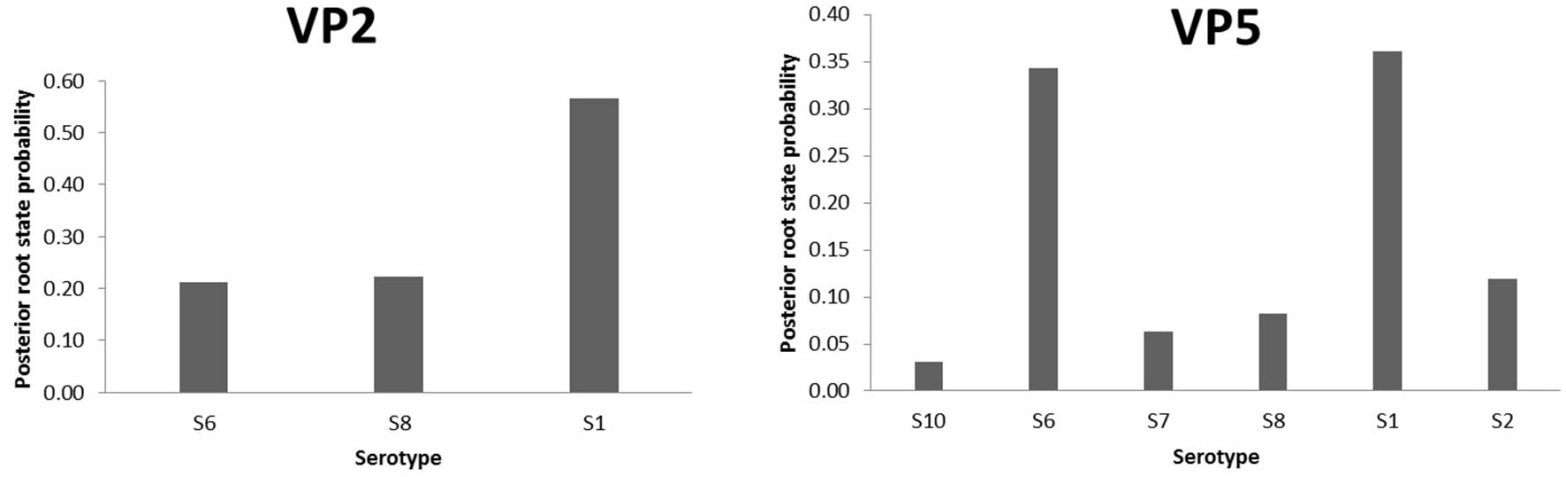
Posterior root state distribution of the “Serotype” model for VP2 and VP5 segments of EHDV.

**Figure 6:**
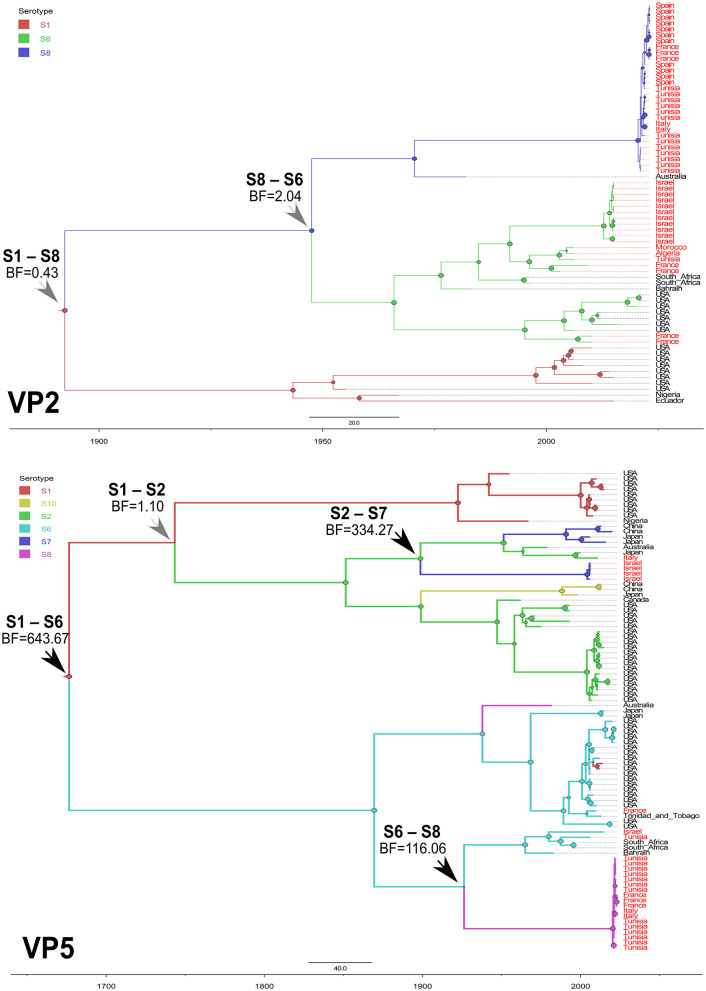
Time-scaled Bayesian MCC trees expressing the viral serotype as discrete trait for VP2 and VP5 segments of EHDV that circulated in the Mediterranean region (Spain, France, Italy, Tunisia, Algeria, Morocco and Israel) during 2006−2023. Black arrows indicate inter-serotype reassortment events related to EHDV of the Mediterranean region, with statistical support (BF > 3). Gray arrows indicate inter-serotype reassortment events related to EHDV of the Mediterranean region, with no statistical support (BF < 3).

To highlight the EHDV epidemiological features about viral ecology and genetic diversity, MCC trees inferring “Serotype” and “Host” (Supplementary Figure S1) traits were generated for VP2 and VP5 segments. For the “Serotype” model, the analysis of root state posterior probability (RSPP) showed that the highest RSPP (RSPP = 0.57) is exhibited by EHDV serotype 1 (S1) based on the VP2 segment dataset. Regarding VP5 segment, the serotypes 1 (RSPP = 0.36) and 6 (RSPP = 0.34) have close values of RSPP and this may indicate that they are the most probable ancestral roots for VP5 segment of the Mediterranean EHDV (Figure 5). VP2 MCC tree analysis also showed that questionable insignificant inter-serotype reassortments (BF = 0.43 for S1–S8; BF = 2.04 for S8–S6) were observed. Noticeably, three out of four inter-serotype reassortment events (S2–S7 and S6–S8) were significantly observed while investigating the VP5 segment. The reassortments S2–S7, S1–S6 and S6–S8 have a high statistical support (BF = 643.67 for S1–S6; BF = 334.27 for S2–S7; BF = 116.06 for S6–S8), whereas, the reassortment between the serotypes 1 and 2 are insignificant (BF = 1.10; Figure 6).

**Figure 7:**
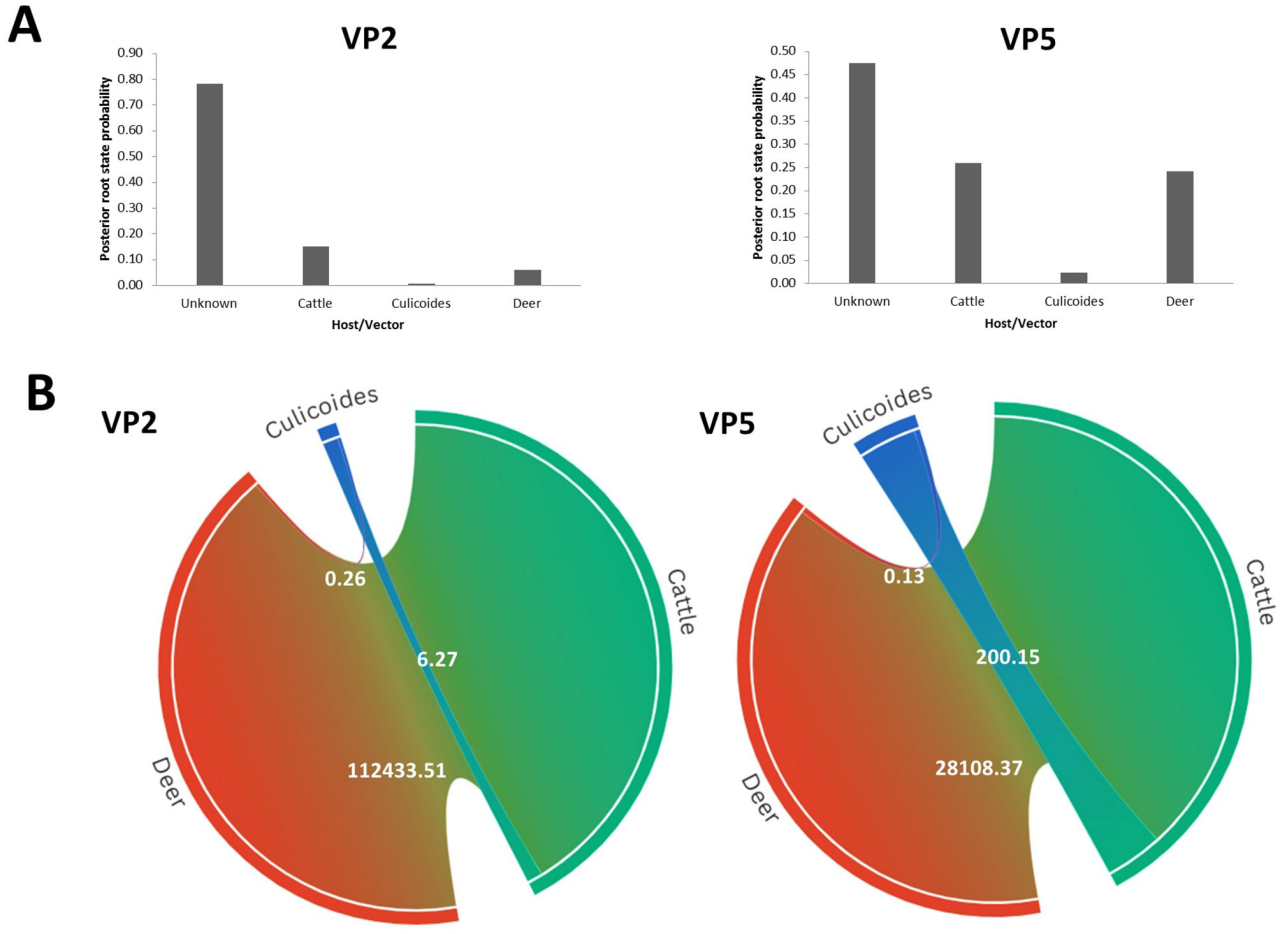
Host phylodynamic analysis for VP2 and VP5 segments of EHDV. **(A)** Posterior root state distribution. **(B)** Chord diagrams showing the transmission routes of virus between the vector (Culicoides) and the hosts (Deer and cattle) with their Bayes factors. BF values are displayed on the chords. Chord width is proportional to BF.

Our phylodynamic analysis also showed that the RSPP values are the highest for the “Unknown” category in the “Host” model of VP2 (RSPP = 0.71) and VP5 (RSPP = 0.48) segments (Figure 7A). As the information about the hosts of the ancestral EHDV strains was not available, the determination of the most probable ancestral host of the Mediterranean EHDV is not feasible with our datasets analysis. However, interpretable results were provided by BF analysis that indicated a strong relationship between EHDV transmission and host/vector types. Indeed, the virus transmission was extremely significant for Cattle–Deer (BF = 28,108.37) and Culicoides–Cattle (BF = 200.15) transition routes of the VP5 segment. For the VP2 segment, the highest BF was detected for Cattle–Deer (BF = 1,112,433.51) transition route and significant BF was also expressed by Culicoides–Cattle (BF = 6.27). For both segments, the virus transmission between Culicoides and deer was statistically invalid (BF = 0.26 for VP2 and BF = 0.13 for VP5; Figure 7B).

### Sampling bias assessment

Based on the tip swap approach, the sampling bias was evaluated to determine the significant transition routes of geographic origin, serotype and host models. The comparison between principal and tip swap analyses showed that the transitions between countries have similar rates or the difference is less than one transition, indicating unbiased results for VP2 and VP5 segments (Table 1). For the “Host” model, the Cattle–Deer and Culicoides–Cattle transitions, in principal and tip swap analyses of VP2 and VP5 segments, expressed similar rates with differences of less than one transition suggesting the absence of sampling bias (Supplementary Table S2). The sensitive analysis of the ‘Serotype' model indicated that the VP5 reassortments S2–S7, S1–S6 and S6–S8 are all unbiased as the difference between their transition rates of principal and tip swap analyses was inferior to one transition (Supplementary Table S3).

**Table 1:** Transition rates and their 95% HPD (in parenthesis) of “Geographic origin” model obtained by principal and tip-swap analyses for VP2 and VP5 segments of EHDV.

**Segment**	**Transition route**	**Principal analysis**	**Tip-swap analysis**
VP2	USA-France	0.89 (8.72 x 10^−5^–2.70)	0.96 (3.49 x 10^−5^–2.93)
	Tunisia–Italy	1.02 (7.30 x 10^−3^–2.57)	0.95 (6.38 x 10^−4^–2.84)
	France–Bahrain	1.02 (1.69 x 10^−4^–2.74)	0.98 (1.05 x 10^−5^–2.87)
	France–South Africa	0.99 (3.94 x 10^−4^–2.77)	1.02 (3.49 x 10^−4^–2.94)
	France–Spain	1.37 (0.04–3.14)	1.02 (2.69 x 10^−5^–2.95)
	France–Israel	0.93 (2.32 x 10^−4^–2.66)	0.95 (5.15 x 10^−5^–2.85)
	Tunisia–Algeria	1.01 (1.55 x 10^−4^–2.71)	1 (1.74 x 10^−4^–2.95)
	Tunisia–Spain	1.86 (0.14–4)	0.97 (1.59 x 10^−4^–2.88)
	Algeria–Morocco	1.03 (9.09 x 10^−5^–2.82)	0.98 (3.11 x 10^−5^–2.87)
VP5	Japan–Israel	1.06 (3.64 x 10^−4^–2.82)	1.01 (1.52 x 10^−4^–2.92)
	Tunisia–Israel	1.09 (5.64 x 10^−5^ –2.97)	1.04 (2.78 x 10^−5^–2.83)
	Tunisia–South Africa	1.05 (1.73 x 10^−5^–2.78)	1.96 (8.82 x 10^−5^–2.96)
	Tunisia–Italy	1.30 (0.01–3.08)	1.01 (9.27 x 10^−4^–2.86)
	Tunisia–France	1.09 (3.28 x 10^−4^–2.88)	0.98 (3.28 x 10^−4^–2.88)
	Japan–Italy	0.98 (6.73 x 10^−5^–2.60)	1 (1.99 x 10^−4^–2.92)
	USA–France	0.80 (2.99 x 10^−4^–2.24)	0.93 (5.43 x 10^−4^–2.84)

## Discussion

In the last decades, the Mediterranean region has become a climate warming hotspot, which had a significant impact on its ecosystems and economics, especially, with the emergence and re-emergence of the viral arthropod-borne diseases such as the Epizootic Hemorrhagic Disease (EHD) (32). Severe clinical signs of EHD have been reported among cattle and deer populations in many countries of the Mediterranean Basin (North Africa, Western Europe and the Middle East) (10–12). The virus spread has caused damage to cattle production, movement bans, trade restrictions, significant expenses for prevention and control measures, and losses of protected deer species in natural reserves (11, 19). Understanding the evolutionary history of such transboundary disease is useful for the control and surveillance of virus emergence in the Mediterranean region. We herein conducted a Bayesian phylodynamic study to gain new insights on the evolutionary history and the epidemiological features of EHDV that circulated in the Mediterranean region during 2006–2023.

As revealed by our study, North America may represent the ancestral root of EHDV in the Mediterranean Basin where the likely first emergence of the virus could have occurred in the 17^th^ century. Similar findings were demonstrated in a phylodynamic study by Alkhamis et al. (29) on the Bluetongue virus (BTV), another arbovirus infecting ruminants. Their study identified a spatio-temporal network of BTV transitions between Asia, Europe, America, and Africa in the 18th and 19th centuries, indicating that European colonization since the 15th century may have increased global livestock trade and contributed to the spread of transboundary diseases. Based on TMRCA estimation, the first introduction of EHDV in the Mediterranean likely occurred in the 1800s, around the same time as the first suspected EHDV cases reported in the USA in 1890 (9).

Our analysis of spatio-temporal dynamics shows that France and Tunisia acted as epicenters for EHDV, facilitating its spread both within and beyond the Mediterranean Basin. The abundance of EHDV vectors and hosts in these countries may have contributed to their role as key geographical points for virus diffusion. Indeed, France is the leading beef producer in Europe (https://sustainbeef.hub.inrae.fr/). It accounted for around 23% of the European bovine population until 2023. (https://ec.europa.eu/eurostat/de/web/products-eurostat-news/-/ddn-20220517-2). Moreover, France ranks second in Europe for Culicoides abundance, following Germany (33), with 45 species of Culicoides midges found to feed on cattle in mainland France and Corsica (34). Similarly, Tunisia's environment provides a suitable ecological niche for Culicoides vectors, potentially facilitating the spread of infected vectors to neighboring North African and European countries (16). Although we did not apply detailed climate models in this study, understanding the influence of environmental conditions on EHDV emergence is an important direction for future work. As a step toward this, using a Generalized Linear Model (GLM) in BEAST could allow us to explore how climate, vector presence, and host populations correlate with viral transmission hotspots. This approach has been already used to highlight the correlation between climatic factors and dispersal of vector-borne viruses notably West Nile virus (35).

In the present study, we also provide evidence of transcontinental EHDV spread, with viral transmission observed between neighboring countries in North Africa (Tunisia, Algeria, and Morocco) and Western Europe (France and Spain). Our findings corroborate other studies that used the Maximum Likelihood (ML) phylogenetic approach to highlight origin of EHDV incursions in Mediterranean countries, based on VP2 and VP5 nucleotide sequences. Indeed, Golender and Hoffmann (12) demonstrated that EHDV strains isolated from Israel between 2015 and 2023 present a strong phylogenetic relationship with strains from African and European Mediterranean countries. Similarly, ML phylogenetic study of Gondard et al. (14) showed that EHDV-8 detected in France in 2023 corresponded to the strain circulating in Tunisia, Italy and Spain since 2021 and 2022. Prevailing winds and illegal livestock trade appear to be key factors contributing to the virus spread in the Mediterranean region. Several studies have demonstrated that winds can disperse infected Culicoides over long distances (36–38), while illegal livestock trade has been reported along the borders of Tunisia with Algeria and in northeastern Morocco, near the Algerian border (39, 40).

Host phylodynamic analysis revealed the highest statistical support for EHDV transmission between cattle and deer, suggesting that the virus circulates in areas with co-habitation of Culicoides, wild ruminants, and domestic livestock, which could facilitate the virus spread. Brown and Morgan (41) highlighted the high prevalence of nematodes among European deer populations that graze alongside cattle, indicating potential transmission of other pathogens between wild and domestic ruminants.

Serotype phylodynamic analysis identified inter-serotype reassortment events (S1–S6, S6–S8, and S2–S7) that marked the evolutionary history of EHDV in the Mediterranean region. The reassortment between S1 and S6 was first reported in Trinidad in 2013, leading to the emergence of the novel EHDV-6 strain in asymptomatic cattle (42). The other reassortment events (S6–S8 and S2–S7) have not been reported before, but the co-circulation of these serotypes in the Mediterranean, the USA, and Japan (8) suggests that the inter-serotype reassortments are possible. As EHD is often asymptomatic in cattle and small ruminants (1, 42, 43), these events may go undetected.

The differences observed between the VP2 and VP5 phylogenetic trees, in terms of the topology of the MCC trees and the inferences of spatio-temporal dynamics, are likely due to a mix of intrinsic viral features and technical reasons. Even though EHDV functions as a single virus, each gene segment can evolve under distinct selection pressures or rates of evolution. The VP2 protein tends to exhibit higher genetic variability compared to VP5 (44). As a result, each gene may reflect a distinct evolutionary history. A major factor behind these differences may also be the uneven availability of sequence data from various countries. In fact, our study is based on publicly available EHDV sequences retrieved from GenBank, which introduce potential biases due to uneven geographic and temporal sampling. Countries such as Tunisia, France, Spain and Israel are better represented, while limited genomic data are available from other Mediterranean regions including Italy, Algeria and Morocco. In addition, no genomic data are available for the other European, African and Asian countries that also compose the whole region of the Mediterranean Basin. Although sensitivity analysis allowed us to evaluate the impact of oversampling on our phylodynamic results, assessing biases related to underreporting remains challenging. Efforts to incorporate additional data from unpublished national veterinary databases or recent field samples could substantially improve the completeness and accuracy of future analyses.

In conclusion, this unprecedented study described the evolutionary history of EHDV in the Mediterranean Basin, including Tunisia, Algeria, Morocco, France, Italy, Spain, and Israel. The findings of this study provide a strong foundation for understanding the phylodynamics and geographic spread of EHDV in the Mediterranean region, which can directly inform animal health strategies. We found that virus transmission was particularly intense between North Africa and Western Europe. Factors such as vector and host distribution, ruminant trade, and prevailing winds likely play a crucial role in EHDV spread within the region, acting as intensifying factors for virus propagation. The observed inter-serotype reassortments reflect the genetic diversity of the Mediterranean EHDV strains. Future research should focus on whole-genome sequencing to enhance the detection of reassortment events and to refine evolutionary insights. Implementing an active surveillance routine of the virus in both wild and domestic ruminants is strongly recommended to identify new serotypes and observe viral dynamics. Additionally, since EHD is asymptomatic in small ruminants (sheep and goats), investigating virus circulation in these species could provide valuable insights into the genetic diversity and ecology of EHDV. Incorporating mathematical modeling that merges ecological, trade, and climate variables could offer valuable forecasts of outbreak risks and guide more effective, targeted intervention strategies amid evolving environmental challenges.

## Data Availability

Publicly available datasets were analyzed in this study. This data can be found at: the data can be queried and download from the genbank.

## Supplementary material

Supplementary Figure S1 Time-scaled Bayesian MCC trees expressing the “viral serotype” as discrete trait for VP2 and VP5 segments of EHDV that circulated in the Mediterranean region (Spain, France, Italy, Tunisia, Algeria, Morocco and Israel) during 2006–2013.

Supplementary Material 1 Metadata of VP2 and VP5 segments of EHDV used in this study.

Supplementary Material 2 KML file describing the spatio-temporal dynamic spread of EHDV based on dataset of its VP2 segment.

Supplementary Material 3 KML file describing the spatio-temporal dynamic spread of EHDV based on dataset of its VP5 segment.

Supplementary Table S1 Number of VP2 and VP5 nucleotide sequences used in this study and their collection year from the Mediterranean countries.

Supplementary Table S2 Transition rates and their 95% HPD (in parenthesis) of “Host” model obtained by principal and tip-swap analyses for VP2 and VP5 segments of EHDV.

Supplementary Table S3 Transition rates and their 95% HPD (in parenthesis) of “Serotype” model obtained by principal and tip-swap analyses for VP5 segment of EHDV.
