# Supplementary material for: Emergence and evolution of epizootic hemorrhagic disease virus in the Mediterranean region: spatio-temporal dynamics and epidemiological insights
Source: Front Vet Sci. 2025 Jul 29;12:1569244. doi: 10.3389/fvets.2025.1569244 (PMC12341390; doi:10.3389/fvets.2025.1569244)
Supplement: Supplementary Table S1 — Number of VP2 and VP5 nucleotide sequences used in this study and their collection year from the Mediterranean countries. [file Table_1.docx]

**Table S1.** Number of VP2 and VP5 nucleotide sequences used in this study and their collection year from the Mediterranean countries

| Country | VP2 | | VP5 | |
| --- | --- | --- | --- | --- |
|  | Number of sequences | Collection year | Number of sequences | Collection year |
| France | 7 | 2003–2023 | 4 | 2010–2023 |
| Italy | 2 | 2022 | 3 | 2011–2022 |
| Spain | 11 | 2022–2023 | 0 | — |
| Tunisia | 14 | 2006–2022 | 14 | 2006–2022 |
| Algeria | 1 | 2006 | 0 | — |
| Morocco | 1 | 2006 | 0 | — |
| Israel | 11 | 2015 | 6 | 2006–2016 |
