# Supplementary material for: Emergence and evolution of epizootic hemorrhagic disease virus in the Mediterranean region: spatio-temporal dynamics and epidemiological insights
Source: Front Vet Sci. 2025 Jul 29;12:1569244. doi: 10.3389/fvets.2025.1569244 (PMC12341390; doi:10.3389/fvets.2025.1569244)
Supplement: Supplementary Table S2 — Transition rates and their 95% HPD (in parenthesis) of “Host” model obtained by principal and tip-swap analyses for VP2 and VP5 segments of EHDV. [file Table_2.docx]

**Table S2.** Transition rates and their 95% HPD (in parenthesis) of ‘Host’ model obtained by principal and tip-swap analyses for VP2 and VP5 segments of EHDV

| Segment | Transition route | Principal analysis | Tip-swap analysis |
| --- | --- | --- | --- |
| VP2 | Cattle – Deer | 1.77 (0.14 -3.91) | 1.24 (6.63x10-4 -3.10) |
|  | Culicoides – Cattle | 0.52 (1.22x10-4 - 1.52) | 0.82 (9.14x10-6 - 2.47) |
| VP5 | Cattle – Deer | 1.59 (0.18 -3.4) | 1.11 (6.70x10-5 -2.83) |
|  | Culicoides – Cattle | 0.57 (0.02 - 1.41) | 0.87 (7.96x10-5 - 2.59) |
