# Supplementary material for: Emergence and evolution of epizootic hemorrhagic disease virus in the Mediterranean region: spatio-temporal dynamics and epidemiological insights
Source: Front Vet Sci. 2025 Jul 29;12:1569244. doi: 10.3389/fvets.2025.1569244 (PMC12341390; doi:10.3389/fvets.2025.1569244)
Supplement: Supplementary Table S3 — Transition rates and their 95% HPD (in parenthesis) of “Serotype” model obtained by principal and tip-swap analyses for VP5 segment of EHDV. [file Table_3.docx]

**Table S3.** Transition rates and their 95% HPD (in parenthesis) of ‘Serotype’ model obtained by principal and tip-swap analyses for VP5 segment of EHDV

| Transition route | Principal analysis | Tip-swap analysis |
| --- | --- | --- |
| S1 - S6 | 1.14 (0.04 - 2.73) | 1.08 (8.72x10-5 - 2.89) |
| S2 - S7 | 1.36 (0.07 - 3.16) | 0.95 (1.41x10-4- 2.67) |
| S6 - S8 | 1.34 (0.06 - 3.13) | 0.95 (4.86x10-5- 2.80) |
