# Supplementary material for: Emergence and evolution of epizootic hemorrhagic disease virus in the Mediterranean region: spatio-temporal dynamics and epidemiological insights
Source: Front Vet Sci. 2025 Jul 29;12:1569244. doi: 10.3389/fvets.2025.1569244 (PMC12341390; doi:10.3389/fvets.2025.1569244)
Supplement: Supplementary Figure S1 — Time-scaled Bayesian MCC trees expressing the “viral serotype” as discrete trait for VP2 and VP5 segments of EHDV that circulated in the Mediterranean region (Spain, France, Italy, Tunisia, Algeria, Morocco and Israel) during 2006–2013. [file Image_1.pdf]

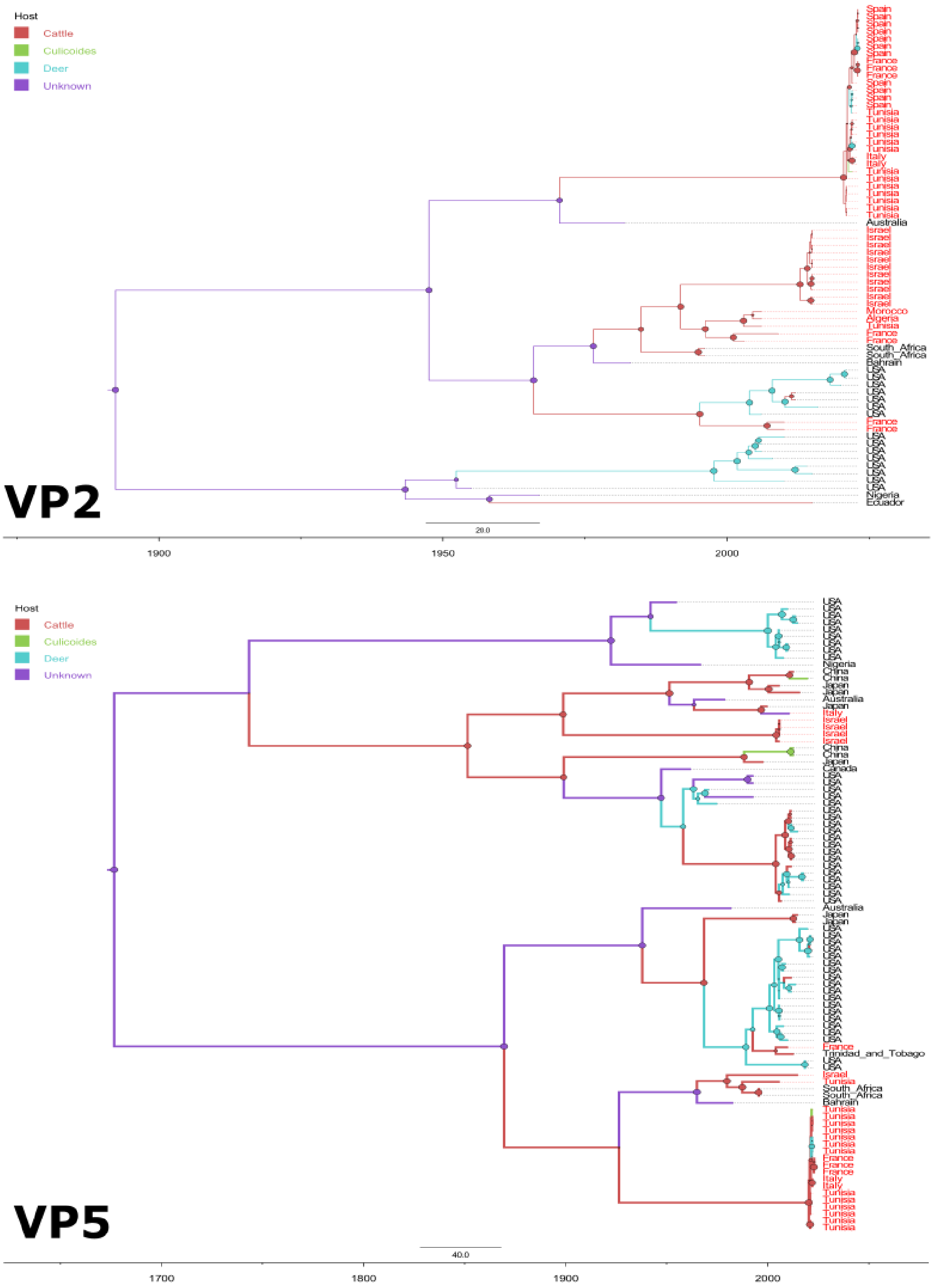

Figure S1. Time-scaled Bayesian MCC trees expressing the ‘viral serotype’ as discrete trait for VP2 and VP5 segments of EHDV that circulated in the Mediterranean region (Spain, France, Italy, Tunisia, Algeria, Morocco and Israel) during 2006-2013.
